# Switching among graphic patterns is governed by oscillatory coordination dynamics: implications for understanding handwriting

**DOI:** 10.3389/fpsyg.2013.00662

**Published:** 2013-09-24

**Authors:** Pier-Giorgio Zanone, Sylvie Athènes

**Affiliations:** Programme Interdisciplinaire de Recherche en Sciences du Sport et du Mouvement Humain, Université de Toulouse, Université Paul SabatierToulouse, France

**Keywords:** coupled oscillators, coarticulation, motor equivalence, self-organization, intention, degrees of freedom, motor theories of perception

## Abstract

Revisiting an original idea by Hollerbach (1981), previous work has established that the production of graphic shapes, assumed to be the blueprint for handwriting, is governed by the dynamics of orthogonal non-linear coupled oscillators. Such dynamics determines few stable coordination patterns, giving rise to a limited set of preferred graphic shapes, namely, four lines and four ellipsoids independent of orientation. The present study investigates the rules of switching among such graphic coordination patterns. Seven participants were required to voluntarily switch within twelve pairs of shapes presented on a graphic tablet. In line with previous theoretical and experimental work on bimanual coordination, results corroborated our hypothesis that the relative stability of the produced coordination patterns determines the time needed for switching: the transition to a more stable pattern was shorter, and inversely. Moreover, switching between patterns with the same orientation but different eccentricities was faster than with a change in orientation. Nonetheless, the switching time covaried strictly with the change in relative phase effected by the transition between two shapes, whether this implied a change in eccentricity or in orientation. These findings suggest a new operational definition of what the (motor) units or strokes of handwriting are and shed a novel light on how coarticulation and recruitment of degrees of freedom may occur in graphic skills. They also yield some leads for understanding the acquisition and the neural underpinnings of handwriting.

## Introduction

A persistent and puzzling issue in motor control is how different motor elemental pieces may be put together into a single unit of behavior. Creating new forms of behavior by combining already existing units is a critical and amazing adaptive ability of living beings on various time scales. Regarding motor behavior, many studies have been devoted to the issue (e.g., Arbib, 1984 or Jeannerod, 1984, on the coordination of the transport and grasping phases in prehension). Yet, as regretfully noted by Schmidt (1988) in the wake of the Schema Theory of learning, the mechanisms and principles through which motor units may be integrated are quite elusive. The converse process of breaking down a whole unit of motor behavior into several separate subunits remains equally mysterious. In particular, it might be necessary to be able to somehow decompose a complex skill into parts in order to isolate the one that is responsible for interference or negative transfer in learning, due to synkinesis or “bad habits,” for instance.

As (motor) development during ontogeny eventually involves putting elemental pieces of behavior together and/or taking them apart, a good deal of work has addressed these issues from a developmental perspective. At a theoretical level, a unique framework is Piagetian theory (e.g., Piaget, 1970): (Motor) schemes, the building blocks of behavior that define its generic structure, can be combined into larger schemes through a process called reciprocal assimilation as they can be dissociated into more specific units as a function of the context through a complementary process called differentiation. White et al. (1964) and Twitchell (1965) provided convincing empirical support for reciprocal assimilation in their study of visually-guided reaching in infants. At the level of gross skills acquisition, phases of dissociation or integration of behavioral units are also observed (e.g., Connolly and Dalgleish, 1989, on spooning), as well as more complicated sequences in which several such phases may occur successively. For instance, Corbetta and Bojczyk (2002) showed that young children switch several times between uni- and bi-lateral hand movements as they acquire the grasping skill, which eventually proves to be a remote consequence of the simultaneous mastering of autonomous walking. In the end, the reaching movements of one-year-old children can be decomposed into a sequence of stereotyped movements each resembling the basic ballistic movements of adults (von Hofsten, 1991; Konczak et al., 1995).

Understanding the integration and dissociation of motor units implies determining their nature and their scope, not only theoretically but also operationally (e.g., Viviani and Cenzato, 1985). A common idea has been that a unit is the part of the motor behavior that is preplanned and can eventually be combined into a more complex unit at a higher level of the motor system (Teulings, 1996, for a review). Several criteria have been devised to identify its boundaries. Probably the most widely accepted definition determines a unit as the segment comprised between two minima of the velocity profile (Brooks et al., 1973). Regarding handwriting, different parts of the written trajectory, such as ballistic strokes (Maarse and Thomassen, 1983), complete allographs (Teulings et al., 1983), upstrokes pairs (Wing, 1978), or even syllables (Kandel et al., 2006, 2011) have all been considered to constitute a graphical unit. Other studies succeeded in extracting small pieces of trajectories—also called strokes—that statistically represent a good part of more complicated handwritten scribbles or letters (Edelman and Flash, 1987; Wada and Kawato, 1995; Wada et al., 1995; Adi-Japha et al., 1998). The definition of a stroke becomes even murkier, since graphical units of cursive handwriting do change as a function of practice (Hulstijn and van Galen, 1988; Lambert and Espéret, 1996), movement velocity (van der Plaats and van Galen, 1991) or context. In fact, it has been shown that when a given parameter of the graphic output is modified, nearly all the other parameters change as well (Thomassen and Meulenbroek, 1993; van Galen and Weber, 1998): Variability affects all graphic variables simultaneously, questioning the very idea of a fixed unit structuring graphic behavior. To the probable dismay of supporters of the notion of stroke, van Galen (1991) conceded that “There is no one, single unit of programming of handwriting; instead, the production unit may depend upon the form of the output” (p. 31).

An altogether different lead into the issue is to conceive of handwriting as the outcome of two combined oscillatory motions, horizontal and vertical, corresponding roughly to the wrist and finger movements, with the addition of a continuous translation (Hollerbach, 1981; Singer and Tishby, 1994), rightward in most writing systems. Basic shapes of cursive handwriting can thus be generated by modulating the values of the relative phase, the relative amplitude and the frequency ratio between the two orthogonal oscillators. However good these models may be at reproducing basic graphic shapes such as loops or straight lines, they fall short of capturing the real behavior of handwriting, that is, producing meaningful 2D trajectories through the coordinated motion of the forelimb and the hand in a behaviorally, biologically and computationally plausible fashion. Two shortcomings will be put forth here. On the one hand, along all prescriptive conceptualizations of motor behavior, these models cannot provide a simple explanation for highly sophisticated motor output, such as handwritten letters and words: the more complex the motor output is, the more complicated the command to the effectors must be. On the other hand, these models fail to address two features specific to handwriting: (a) the presence of preferred graphic shapes and directions that constrain the production of a script (e.g., van Sommers, 1984) and determine how it deteriorates under speed constraints (e.g., Dounskaïa et al., 2000); and (b) the coarticulation between shapes, that is, the pro- and retroactive alterations of a graphic unit with respect to the following and preceding ones.

An entry point into the above issues is a dynamic approach to coordination (Schöner and Kelso, 1988; Kelso, 1995), especially regarding trajectory formation. Planar trajectories proved to result from coordinating the periodic motion of two orthogonal non-linear oscillators (Buchanan et al., 1996, 1997; De Guzman et al., 1997). Not only does such coordination give rise to different stable trajectories, such as 1, 0, C, or 8, as a function of the respective parameters of the two oscillators, but an orderly transition between the shapes (from 8 to 0 to 1) occurs as a given coordination loses stability with increasing speed. In line with the principles of the approach developed after the seminal work by Kelso (1984) on bimanual coordination, stability is both a key concept for understanding coordination and a key property of behavior for predicting its evolution under various levels of constraints, because stability rules the maintenance and the change of coordination patterns.

Extending this idea to handwriting, previous work (Athènes et al., 2004) showed that in a simple task requiring the production of different graphic shapes, only a few among those were spontaneously performed in a stable and precise fashion, namely, a line and an ellipsoid of intermediate eccentricity in four different orientations. These preferred shapes corresponded to specific and stable phase relationships between two frequency-locked orthogonal non-linear oscillators. Theoretically, these relative phases define stable states or attractors of the dynamics underlying handwriting. The presence of attractors is revealed by systematic distortions that handwriting exhibits toward these preferred orientations and shapes, especially under a high level of constraints, such as writing at high speed or using a non-dominant hand (Sallagoïty et al., 2004). In turn, such preferred shapes are marginally distorted by the influence of “low-level” constraints such as the biomechanical properties of the end effector, here the difference in excursion and frequency between the oscillators (Danna et al., 2011). In summary, like most if not all periodic motion, graphic skills, and probably handwriting, are governed by the dynamics of non-linear coupled oscillators, which determines how they are produced, how they adapt, and how they deteriorate. Based on these premises, we have proposed a parsimonious oscillatory model that simulates handwriting quite efficiently, from so-called basic strokes to quite complicated individual signatures to complete sentences (André et al., in revision).

A first outcome of the above work is a novel definition of a behavioral unit of writing: A unit is a segment of the trace that corresponds to a specific and stable value of relative phase, namely, about 0°, 60°, 120°, or 180° (Athènes et al., 2004). Different letters in succession, such as “e” and “l,” for instance, might pertain to the same unit, because they involve the same relative phase pattern. In contrast, a single letter, such as “g,” implicates several units, because different phase relationships are necessarily implemented in succession in order to realize the required trajectory. Now, a step toward a more comprehensive model of handwriting is to establish the rules of passage between such “dynamic” graphic units, that is, the switching between underlying attractors. A principle, well established theoretically and empirically, is that stability governs how the switching among coordination patterns occurs (Kelso et al., 1988; Scholz and Kelso, 1990; Carson et al., 1994): a transition to a fairly stable pattern will take less time than a transition to a less stable pattern.

The present study aims to assess the switching time, τ_sw_, among the four stable graphic coordination patterns identified in our previous work. We assumed that τ_sw_ between two patterns is determined by their relative stability, according to the order of stability between the attractive patterns reported in previous studies (Athènes et al., 2004): 0° is more stable than 180°, which in turn is more stable than 60°, which is more stable than 120°. Thus, for all transitions within pairs of patterns, the time incurred to intentionally switch from a more stable pattern to a less stable pattern will be significantly larger than the other way round.

## Material and methods

### Participants

Seven right-handed participants (five male, two female) aged 25–42 (mean 29.1; *SD* = 6.0) volunteered for the experiment. Hand dominance was determined by a questionnaire of hand preference in various daily tasks (Dellatolas et al., 1988). They all reported normal or corrected-to-normal vision and were naive to the purpose of the experiment. Each participant filled out a written consent form before the experiment. The experimental protocol received full approval from the local ethics committee according to the Helsinki convention.

### Task

**Figure 1:**
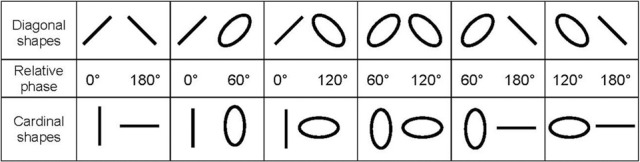
**The 12 pairs of shapes in the two orientations used in the switching task with their respective relative phase value.** The 0°/180° and 60°/120° relative phases correspond to shapes with 0.00 and 0.82 eccentricities in the two orthogonal orientations, respectively.

Participants were required to reproduce two shapes displayed consecutively on a digitizing tablet placed in front of them using an attached stylus. Instructions were to be as accurate as possible at a constant speed and to always maintain contact between the stylus and the writing surface. The shapes differed relative to their basic orientation, diagonal or cardinal, and their eccentricities, defined by the relative phase between the orthogonal oscillators used to produce them (see Figure 1, for details). In a first Period (P1), an initial shape was produced for 4 s, corresponding to 15 cycles, since performance was paced by an auditory metronome at a frequency of 3.75 Hz, the average value exhibited spontaneously in our previous study (Athènes et al., 2004). Then, in a second Period (P2) lasting also 4 s, 15 additional were produced without metronome but at the same frequency. Finally, in a post-switching period (PS), the initial shape displayed on the screen was replaced by a different one, which was to be reproduced for 8 s (viz. 30 cycles) still without pacing signal. Participants had to switch from the first to the second shape as fast as possible, without attempting to anticipate the moment of switching. For each pair shown in Figure 1, participants had to switch between shapes in both directions (e.g., 0°–180° and 180°–0°). Henceforth, Direction 1 (D1) implies a transition from a stable to a less stable pattern, and inversely for Direction 2 (D2).

### Procedure

For each participant, the experiment lasted for 2 h. At the beginning, participants were familiarized with the apparatus and the task. They were asked to draw a circular shape for 30 s in a counterclockwise fashion and to perform three exemplary transitions with the cardinal and the diagonal set of shapes. The position of the wrist on the tablet that was adopted on the first trial was recorded by pasting a marker on the surface, so that the wrist could be set similarly in subsequent trials. Then, participants had to perform six repetitions of the 12 possible transitions in both directions, with a random assignment of the required relative phases to each trial.

### Apparatus

All shapes, inscribed within a circle of 2 cm of diameter, were displayed at the center of a 21 × 15 cm backlit digitizing tablet (Wacom PL300), which was inserted in a table of adjustable height facing the participants. As soon as the stylus touched the tablet, the *x* and *y* coordinates (accuracy = 0.50 ± 0.02 mm) of the performed trajectories were digitized at 100 Hz, fed back on-line for display on top of the current model shape on the tablet, and stored for later processing.

### Data processing

**Figure 2:**
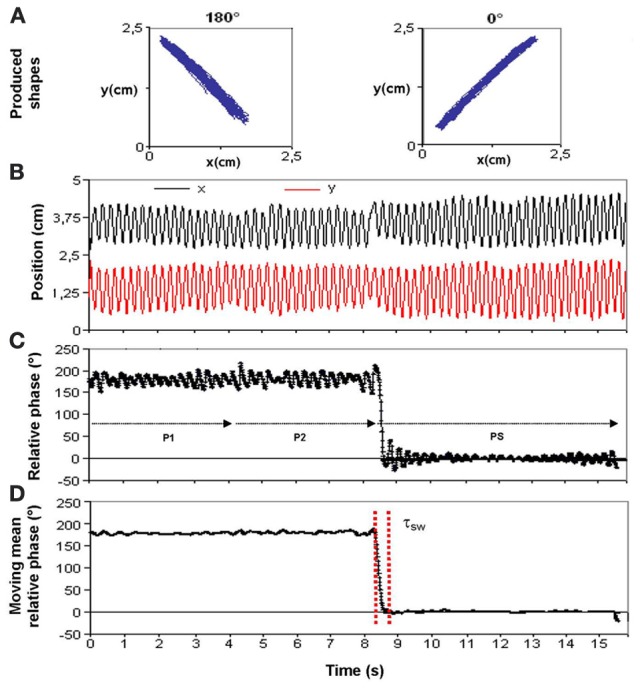
**Measurement of switching time for a typical D1 transition from 180° to 0° of relative phase (see text for details)**.

Figure 2 displays one trial for a single participant for the sake of illustrating the various steps involved in getting an accurate assessment of switching time, τ_sw_, the time needed to change pattern. Specifically, Figure 2 reports a transition from 180° to 0°, a typical D2 transition, from a less stable to a stable pattern, occurring between the periods before and post switching (P2 and PS). Panel A shows the two traces actually produced on the digitizing tablet. Panel B displays the time series of the *x* and *y* components of the traced trajectory. Panel C presents the continuous relative phase in degrees between those oscillatory components calculated through a Hilbert transform routine (see Rosenblum et al., 2001, for details). The successive periods of the experimental procedure (P1, P2, and PS) are also denoted. Panel D shows the continuous relative phase averaged within a moving window spanning the mean cycle duration over a trial. Switching time (in ms) was defined between the moment at which performance exited the stability region of the current relative phase pattern and the moment at which it entered the stability region of the new pattern. The criterion for a stability region was set at ± 2 *SD* about the current mean value for a cycle of performance. In Figure 2, the transition between 180° and 0° occurred at about 8.5 s and lasted 354 ms, expressed as the time interval comprised between the two vertical red dotted bars defining the exit from and entry to the stability region. Then, the Absolute Error (AE) and the Standard Deviation (SD) of relative phase were calculated in order to assess the accuracy and stability of the produced pattern, respectively, in the pre- and post-switching periods (P2 and PS).

## Results

Results will be presented in three subsections. First we analyze how each individual component (*x* or *y*) behaves over the entire procedure, especially in terms of the stability of their oscillations as a function of the three periods of the experimental procedure (P1, P2, and PS). Second, we consider their collective behavior, namely, the stability of the relative phase between the oscillations of the components. Finally, we examine the properties of the switching between pairs of shapes, in order to test our main hypotheses.

### Component stability

In order to assess the stability of oscillation, we used the *SD* of the period of each component, the mean value being close to the inverse of the required frequency 3.75 Hz. We compared the variability of the produced period by the two oscillators between the pre- and post-switching periods (P2 and PS, respectively) and over the four required relative phases (0°, 60°, 120°, and 180°) for each set of shapes separately (cardinal and diagonal).

For each set, an Oscillator (2) × Pattern (4) × Period (2) ANOVA with repeated measures on all factors revealed a main effect of Period, *F*_(1, 6)_ = 79.04; *p* < 0.05 and *F*_(1, 6)_ = 40.51; *p* < 0.05, for the diagonal shapes and the cardinal shapes respectively. For all patterns and transitions, the variability of the component period of oscillation was significantly lower for the pre-switching than the post-switching period. However, results did not reveal any difference in variability of the oscillation period as a function of the pattern produced. This finding rules out the possibility that variations in the stability of the relative phase between the *x* and *y* components due to their coupling as a function of the produced relative phase be a mere result of variations in the periodic behavior of the individual oscillators.

### Collective stability

We compared the accuracy and the variability of the produced relative phase between the pre- and post-switching periods (P2 and PS, respectively) and over the four required relative phases (0°, 60°, 120°, and 180°) for each set of shapes separately (cardinal and diagonal).

**Figure 3:**
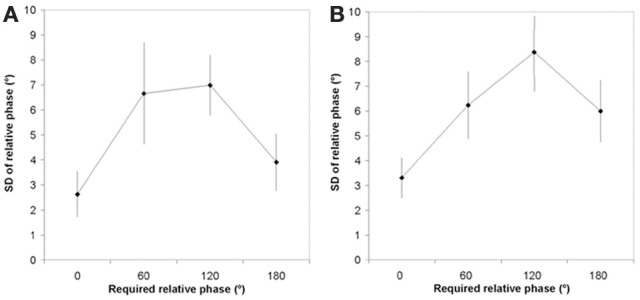
**Standard deviation of the produced relative phase as a function of the required relative phase for the diagonal shapes (Panel A) and the cardinal shapes (Panel B).** Vertical bars correspond to inter-participants *SD*.

Two separate Pattern (4) × Period (2) ANOVAs with repeated measures on both factors were performed on the AE and the SD and of the relative phase for the two sets of shapes. No effect turned out to be significant for the AE of relative phase in any set. In contrast, the ANOVA revealed a significant Pattern effect for the diagonal shapes, *F*_(3, 18)_ = 48.46, *p* < 0.0001, and the cardinal shapes, *F*_(3, 18)_ = 57.61, *p* < 0.0001. Figures 3A,B display the SD of the relative phase as a function of the required relative phase for the diagonal and cardinal shapes, respectively. On the one hand, they show that the variability of relative phase was basically comparable to those reported in our previous studies, ranging between 3°and 9° (e.g., Athènes et al., 2004). On the other hand, they display similar trends as a function of the required relative phase, with, as expected, 0° and 180° being more stable than the other relative phases. *Post-hoc* Newman-Keuls tests indicated that the 0° pattern was the most stable, while the 180° pattern was more stable than 60°, which in turn was more stable than 120, all *ps* < 0.01. This is important, because the relative stability of the patterns determines the switching time needed to go from one shape to another. Note that the error in producing the required relative phases followed the same order: The most stable patterns were also the most accurate, a finding already reported in our previous studies on bimanual coordination (Kostrubiec et al., 2006; Tallet et al., 2008) or on graphic skills and handwriting (Danna et al., 2011).

### Switching time

**Figure 4:**
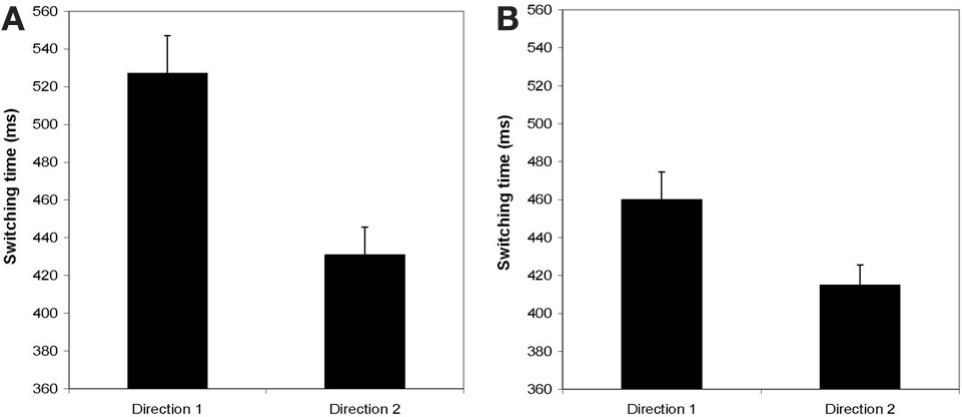
**Average switching time as a function of the Direction of the transition for diagonal shapes (Panel A) and for cardinal shapes (Panel B).** Direction 1: from a stable to a less stable pattern; Direction 2: from a less stable to a stable pattern. Vertical bars correspond to inter-participants *SD*.

Figure 4 displays the averaged switching time, τ_sw_, as a function of the Direction of switching for the diagonal (Panel A) and the cardinal shapes (Panel B). Results revealed that irrespective of the patterns involved in the transition, switching from a less stable to a more stable pattern (Direction 2) was significantly shorter than the other way round (Direction 1). The mean difference was 97 ms, *F*_(1, 6)_ = 13.82, *p* < 0.01, for the diagonal shapes, and 35 ms, *F*_(1, 6)_ = 5.03, *p* < 0.01, for the cardinal shapes. Another finding was that switching between cardinal shapes (Figure 4B) was faster, τ_sw_ = 435, than between diagonal shapes (Figure 4A), τ_sw_ = 4.0 ms.

**Figure 5:**
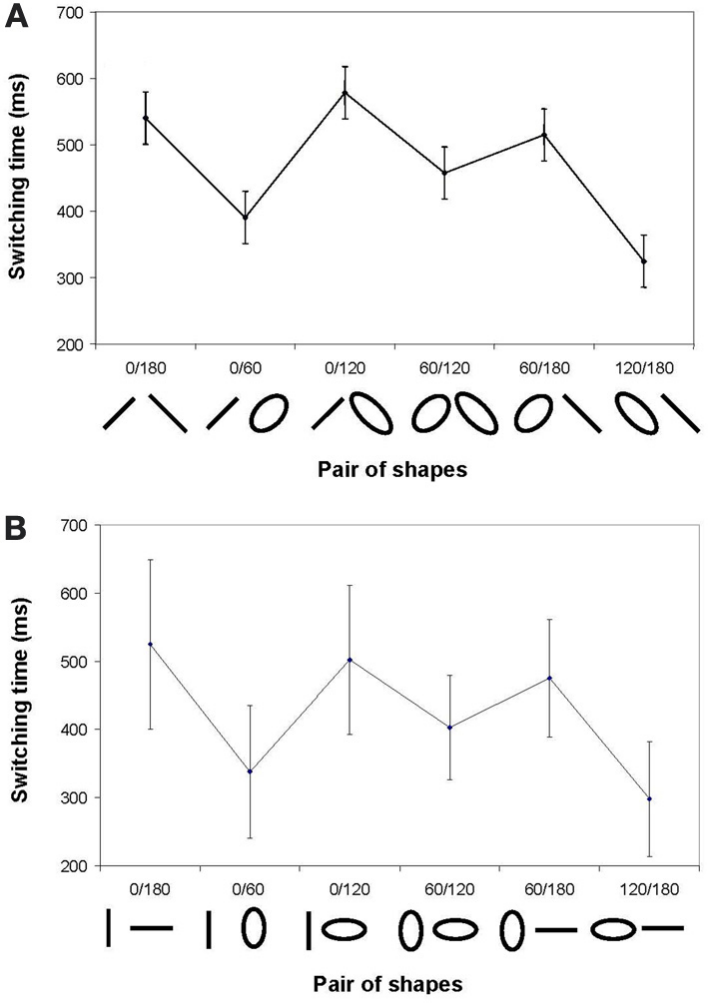
**Average switching time between pairs of diagonal shapes (Panel A) and cardinal shapes (Panel B).** Vertical bars correspond to inter-participants *SD*.

Figure 5A displays the average switching time, τ_sw,_ as a function of the pairs of diagonal shapes involved in the transitions, irrespective of the direction (D1 and D2 are pooled). A Pair (6) ANOVA with repeated measures reveals a significant effect, *F*_(5, 30)_ = 10.37, *p* < 0.0001. A *post-hoc* analysis showed that the switching time for 0°/60° and 120°/180° transitions (358 ± 47 ms) were similar and significantly shorter than for 0°/180°, 0°/120°, 60°/180°, and 60°/120° transitions (522 ± 50 ms). Moreover, switching times for 0°/180°; 0°/120°, 60°/180°, and 60°/120° did not differ, with a single exception for 60°/120° that was significantly shorter than 0°/120°. These findings indicate that switching between diagonal shapes that were in the same orientation but with different eccentricities (i.e., 0°/60° and 120°/180°) was faster than for shapes that required a change in orientation.

Figure 5B displays the average switching time, τ_sw_, as a function of the pairs of cardinal shapes involved in the transitions, D1 and D2 pooled. A Pair (6) ANOVA with repeated measures reveals significant effect of Pair, *F*_(5, 30)_ = 5.17, *p* < 0.001. By and large, Figure 5B is very similar to Figure 5A, except for the generally lower average values already reported above. *Post-hoc* analyses confirm that transition between 0°/60° and 120°/180° (327 ± 36 ms) were faster than the others (472 ± 42 ms), with transitions between 60°/120° differing from those between 0° and 180°. Here again, switching between cardinal shapes that were in the same orientation but with different eccentricities was faster than for shapes that required a change in orientation.

**Figure 6:**
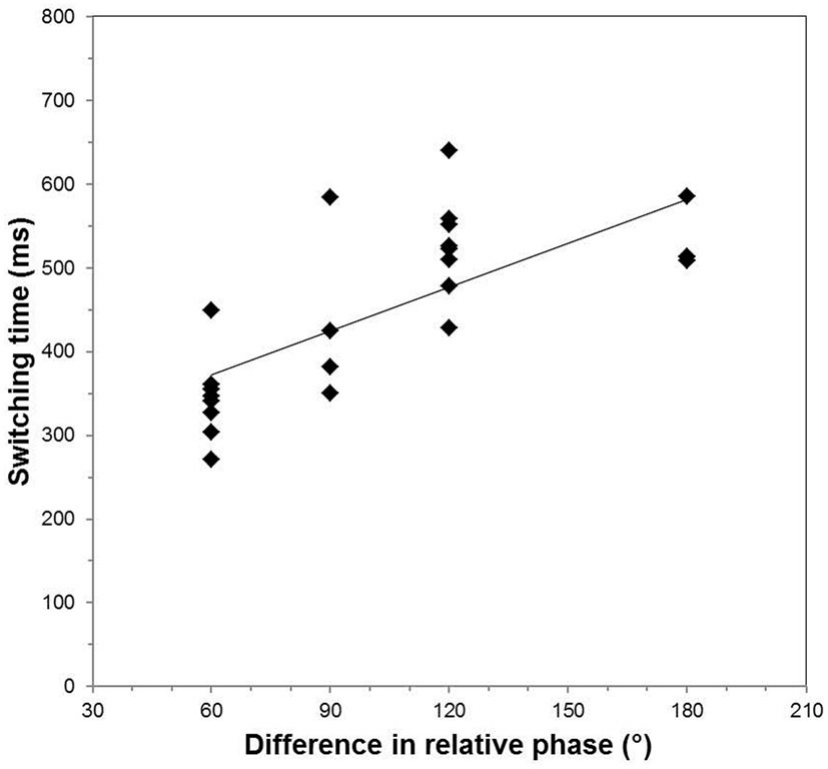
**Overall switching time as a function of the required change in relative phase, irrespective of the orientation and the eccentricity of the required shapes**.

The latter effect, valid for both orientations, can be further exposed by analyzing the switching time necessitated to perform a given change in relative phase required by the task, irrespective of the actual shapes to be produced, namely, its shape and orientation. Figure 6 presents the average τ_sw_ as a function of the absolute difference between the starting and final relative phases composing all the pairs of shapes. In such a display, τ_sw_ grows linearly with the difference in relative phase (*R*^2^ = 0.551, *p* < 0.01). Whether due to a change in eccentricity or in orientation or both between the two required shapes, the larger the change in relative phase is, the longer it takes to perform the switching.

## Discussion

In line with a dynamic approach to coordination, the present study confirms that stability is a theoretical concept and an empirical property of coordinated behavior. In particular, stability is crucial to understanding the production of fairly simple graphic shapes, namely ellipsoids, which are conceived of as basic primitives for real handwriting (Athènes et al., 2004). Indeed, stable coordination patterns correspond to the most frequent and precise graphic shapes (Athènes et al., 2004), which resist better deleterious constraints (Sallagoïty et al., 2004), entail deformations in realizing other required shapes (Danna et al., 2011), and are produced fastest (Athènes et al., 2004) as well as with the lowest attentional load (Kostrubiec et al., in press). The present study adds another interesting if expected property: The stability of a graphic coordination pattern also determines the switching time therefrom and thereto, so that the more stable a pattern is, the less time it takes to implement it and the more time it demands to switch to another pattern. As relative phase proves to be a variable that captures both the produced coordination pattern and the resulting shape that is traced, this rule specifies how swiftly a writer can switch between the various corresponding shapes (cf. Figure 1), which may differ in orientation and eccentricity.

This ensemble of coherent findings about the coordination dynamics underlying graphic skills is quite in line with what is generally known about coordinated oscillatory movements, in particular with the bulk of research accumulated on interlimb coordination. A first consequence is that the coordination dynamics subserving graphic skills, and most probably handwriting, are basically compatible with general theories of coupled oscillators, a prominent instance of which is the HKB model (Haken et al., 1985) and its numerous avatars (e.g., Peper et al., 2008). In particular, the present paper corroborates that the non-linear coupling between the oscillating components determines the stability of the produced coordinated pattern, irrespective of the stability of the individual components, a finding already reported for bimanual coordination (Temprado et al., 1999).

In the same vein, a challenging finding is that transitions that do not necessitate a change in orientation (viz. that require only a change in eccentricity) are always shorter than transitions between shapes of different orientations. Previous work on 2D trajectory formation (e.g., Buchanan et al., 1997; Calvin et al., 2004) —a general framework for handwriting indeed—proposed that recruiting or annihilating a degree of freedom over the ongoing oscillatory motion of a given joint (e.g., passing from 0° to 60°, and vice-versa, a change in eccentricity, cf. Figure 5) is less involved than changing the temporal organization of the motor command altogether, such as switching the two joints motion on and off alternately (e.g., passing from 0° to 180° or vice-versa, a change in orientation). However, seductive this biomechanically-oriented view is regarding the production of the diagonal shapes (Serrien and Swinnen, 1997, 1999), which are fairly aligned with the motion of the wrist and fingers, it does not hold for cardinal shapes (cf. Figure 4), the production of which always combines the activity of both “physical/biomechanical” oscillators. A question arises whether the recruitment of degrees of freedom issue is specifically irrelevant regarding handwriting, perhaps because it is a highly practiced skill, or whether it is an insoluble question regarding all trajectory formation processes, and coordinated motor behavior for that matter.

But what does this better knowledge on the effects of stability on switching time bring into our understanding of handwriting?

First, the present results corroborate previous evidence that the dynamical properties of the graphic patterns are largely independent of their orientation: for cardinal and diagonal shapes as well, variability of relative phase and switching time are confined within the same range and show the very same effects as a function of the required relative phase (viz. the shape to be traced). Yet, producing the same graphic shapes in various orientations implies an altogether different mobilization of the joints and muscles of the hand and the wrist. Simply put, it involves radically different motor commands. Therefore, in a dynamic framework, the two orthogonal coupled oscillators constitute an abstract model “living” in the space of relative phase—and probably somewhere in the brain, as an outcome of the collective, synchronized activity of the neurons—as opposed to a more physical or applied model *à la* Hollerbach, in which the oscillators stand for real, if simplified, end effectors (fingers and hand) periodically moving about joints. Such independence of coordination dynamics from the effectors, a property already reported in Kelso and Zanone (2002), may be at the origin of the well-known motor equivalence characterizing handwriting (Merton, 1972), that is, the ability to achieve the same motor output through various combinations of muscle and joint activities. Of course, the actual trace will exhibit lawful deformations with respect to the canonic exemplar, as a function of orientation, reflecting the differential influence of the properties (e.g., biomechanical) of the actual oscillators on the produced trajectories. Example of such surface effects have been reported in Danna et al. (2011). As well, other deformations due to other constraints on the trace production, such as the writing posture, handedness, or the paper or pen characteristics, are likely to occur and still await assessing.

Second, handwriting speed is not only dependent on how many changes in relative phase are required for producing a given trace, each demanding a given amount of time, but also on how big such jumps are. The tight relationship reported here (Figure 6) between the change in relative phase and the associated switching time is a novel finding leading to three lines of thought. Firstly, it is reminiscent of many effects in psychophysics or cognitive sciences in which the duration of a mental or motor operation is proportional to one of its spatial parameters. A famous instance is the linear covariation of the time taken for mentally rotating an object and the angle of rotation (e.g., Shepard and Cooper, 1982, for a review), suggesting that rotation is performed at a constant “mental” speed. In the case of handwriting, an analogy could be tempted. A big jump in relative phase, which corresponds to a large change in eccentricity or in orientation, leads to a sharply bended trajectory. Thus, the more curved the trajectory, the more time is necessitated, as though there were a constant “speed of bending.” Such a tentative effect might represent a qualitative reflection of the 2/3 power law relating the tangential velocity to curvature (Lacquaniti et al., 1983). Secondly, the covariation between switching time and the jump in relative phase implies that for a trace of a comparable length involving the same number of changes, the larger the jumps are, the slower the trace is. Thus, maintaining the trace within a narrow range of changes in relative phase about some central values is a tentative mechanism for increasing speed. Although, to our knowledge, no extensive data is available regarding the distribution of the relative phases produced during handwriting, odds are high that only a limited subset is performed frequently, thereby narrowing the range of possible jumps. Interestingly, the replacement of a 90° stable coordination pattern with two new ones located at 60° and 120° that underlies the evolution from a round childish handwriting to the spikier slanted adult one (Danna et al., 2012) may just realize such a decrement in the size of the possible jumps in relative phase values, which may concur in part to a concomitant increase in speed. Still, this effect of the jump in relative phase on the overall writing speed is but an additional contribution, since the stability of the performed coordination pattern itself proves to substantially affect the associated attentional load, hence the speed allowed for drawing a given shape (Kostrubiec et al., in press). Thus, the difficulty of the writing task is a complicated function of the shape to be produced, which can be nonetheless sorted out in terms of the dynamic properties of the coordination patterns implemented. Thirdly, the relationship between switching time and the size of the change in relative phase provides a glimpse into a(nother) reason why handwriting transforms with increasing speed. Not only some relative phase patterns—the least stable ones to start with—destabilize completely, which renders the corresponding shapes altogether unfeasible (Sallagoïty et al., 2004), but those patterns that remain marginally stable may be just “unreachable,” because the time it would take to switch to them is too long within the speed constraints. Only patterns that are still “switchable to” in a fair amount of time remain accessible, so that they eventually replace the intended ones, which induces deformations in the performed trajectory.

A third general lesson is that knowing how transitions between graphic units occur provides a key for understanding coarticulation, a phenomenon typical of speech (e.g., Liberman et al., 1957; Benguérel and Cowan, 1974; for a review see Galantucci et al., 2006), typed language (e.g., Viviani and Laissard, 1996) as well as written language (e.g., Thomassen and Schomaker, 1986; van Galen et al., 1986). Regarding cursive handwriting, coarticulation refers to the forward- and backward influence between two successive graphic units, which can smudge into each other (Sosnik et al., 2004) or be connected by an additional segment of trajectory (Meulenbroek and van Galen, 1989). Such variations in trajectory due to coarticulation turn out to inform on the forthcoming letter to be written: the kinematics of the letter being produced allows for a reliable anticipation of what letter is to be traced next (Kandel et al., 1994). Such perceptual prediction works equally well whether the current letter is part of a simple diagram or of a full word. Irrespective of any other movement parameter, the second letter can be reliably predicted by viewing only a constant fraction (the final 60%) of the downstroke of the first letter. A nice extension of the effect to trigrams suggests that slight modulations of the basic 2/3 power law linking tangential velocity to curvature (Lacquaniti et al., 1983) provides cues for anticipating the forthcoming letter (Kandel et al., 2000).

In view of the evidence provided above, our conviction is that relative phase may well be a(nother) variable that carries information about where the trajectory being traced is heading next: A slight modulation about a stable relative phase might be a clue for a forthcoming switch to another relative phase. Indeed, the visual system proves to be pretty sensitive to variation in relative phase in various dynamic displays (Zaal et al., 2000), but also in static displays such as a trace (Wamain et al., 2011). Moreover, discrimination is all the more efficient that the relative phase pattern to be discerned from is stable, a finding already reported in early experiments on learning novel relative phase patterns (Zanone and Kelso, 1992). In particular, any change from straight lines (0° or 180°) or from pieces of curves corresponding to intermediate ellipses (60° or 120°) are likely to be most evident and provide cue for a forthcoming jump to another stable value of relative phase. This assumption is worth an empirical confirmation.

Finally, the foregoing arguments convey an implicit conjecture that producing and perceiving relative phase coordination patterns and their outcome on the paper share common processes, or dynamics as we would say. There is growing evidence, gathered first in the field of speech science in the fifties (e.g., Liberman and Mattingly, 1985, for a review), supporting the idea that perception is strongly rooted into action, a basic tenet of so-called “motor theories of perception” (e.g., Galantucci et al., 2006, for review). Many behavioral and neurobiological findings indicate that such is the case for handwriting too (e.g., Nakamura et al., 2012). In particular, in the wake of the “mirror neurons” discovery, studies using fMRI have shown that reading handwritten letters involved areas in the prefrontal cortex that were common with the network activated in reading the same letters, whereas this was not the case for printed letters or scribbles (Longcamp et al., 2003). A finer analysis using ERP demonstrated that the involvement of the motor cortex in the discrimination of letters is fairly precocious, as early as 300 ms after the stimulus (Wamain et al., 2012). Now, in the specific task of discriminating between ellipses of various eccentricities, a recent study using a dual-task paradigm evidenced that a concurrent motor task was interfering with visual perception exclusively for ellipses that were actually produced in the most stable fashion for a given individual (Wamain et al., 2011). Thus, the motor areas subserving the movement of the hand are significantly more involved in detection and discrimination for shapes that correspond to stable coordination patterns, as compared to shapes corresponding to less stable ones. For the time being, nothing is known about the neural underpinnings of switching among several relative phase patterns. Nonetheless, it has been shown that a specific stimulation on the Supplementary and Premotor cortices by TMS can trigger a change between two stable bimanual patterns (Meyer-Lindenberg et al., 2002), more specifically from the less stable anti-phase to the more stable in-phase pattern. Although the mechanism involved in that case, linked to perturbation and loss of stability in the network, is probably not the same, this finding fathoms that an analysis of voluntary switching is open to investigation at the neural level.

In the same line of thought, one has to keep in mind that handwriting is a complex skill that goes beyond the coordination dynamics explored here, which accounts for the lawfulness of the generation process of the graphic trace. For sure, the establishment of any efficient writing system must have implied a coevolution with the corresponding reading system. Thus, the preferred motor output determined by underlying coordination dynamics has been co-defined by the perceptual categories that discriminate between the visible traces to form a coherent and meaningful perceptual pattern. This has certainly been a long process through mankind's history (e.g., Changizi and Shimojo, 2005). It is also the case on the ontogenetic scale: Reading and writing are among the lengthiest to acquire in children, and oftentimes troubles in both skills are definitely intermingled.

### Conflict of interest statement

The authors declare that the research was conducted in the absence of any commercial or financial relationships that could be construed as a potential conflict of interest.
